# Biomarkers for Screening and Diagnosis of Heart Failure in Cardiovascular–Kidney–Metabolic Syndrome: A Narrative Review

**DOI:** 10.3390/ijms27052462

**Published:** 2026-03-07

**Authors:** Anda-Maria Pintea, Ioan-Alexandru Minciună, Dana Pop

**Affiliations:** 1 Rehabilitation Cardiology Discipline, 4th Department of Internal Medicine, “Iuliu Hațieganu” University of Medicine and Pharmacy, Victor Babeş Street, No. 8, 400012 Cluj-Napoca, Romania; anda.mari.pintea@elearn.umfcluj.ro; 2 Doctoral School, “Iuliu Hațieganu” University of Medicine and Pharmacy, Victor Babeş Street, No. 8, 400012 Cluj-Napoca, Romania; 3 Cardiology Department, Clinical Rehabilitation Hospital, Viilor Street, No. 46-50, 400347 Cluj-Napoca, Romania

**Keywords:** cardiovascular–kidney–metabolic syndrome, heart failure, prediction score, biomarker

## Abstract

Cardiovascular–kidney–metabolic syndrome is a novel concept defined by the American Heart Association, highlighting the complex interactions between the cardiovascular system, kidney function and metabolic risk factors. Poor cardiovascular–kidney–metabolic health is increasingly prevalent worldwide, giving rise to a need to optimize early detection of cardiovascular dysfunction. Heart failure is one of the most prevalent forms of cardiovascular disease in patients with chronic kidney disease and metabolic risk factors, but screening and diagnostic strategies remain challenging. Current guidelines endorse the use of prediction scores, as well as a biomarker-based strategy in patients at increased risk. Despite evidence supporting the use of biomarkers such as natriuretic peptides, there are considerable limitations to their use in the setting of cardiovascular–kidney–metabolic syndrome. Moreover, there is mounting evidence supporting the use of other biomarkers reflecting underlying mechanisms leading to heart failure. The aim of this review is to assess current approaches to screening for and diagnosing heart failure in cardiovascular–kidney–metabolic syndrome, highlighting the strengths and pitfalls of gold-standard and emerging biomarkers, while also addressing gaps in evidence and future research directions. Validation of screening biomarkers and development of multimarker prediction scores could impact clinical practice and reduce the growing morbidity and mortality in cardiovascular–kidney–metabolic syndrome.

## 1. Introduction

**Figure 1:**
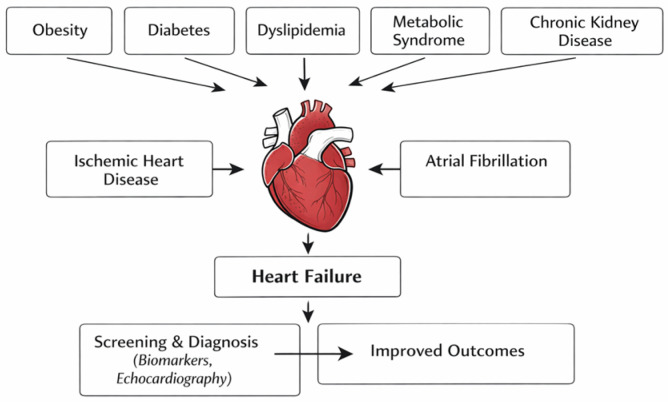
Cardiovascular disease in cardiovascular–kidney–metabolic syndrome.

The novel concept of cardiovascular–kidney–metabolic (CKM) syndrome defined by the American Heart Association (AHA) has led to a change in paradigm regarding prevention, screening and therapeutic approaches of cardiovascular disease (CVD) [1]. Given advances in understanding the complex interactions between metabolic risk factors and kidney dysfunction in the pathogenesis of CVD, the framework of CKM syndrome highlights the need for an integrative, multidisciplinary approach. As per the definition proposed by the AHA, CKM syndrome includes patients with established CVD related to kidney dysfunction and metabolic risk factors such as excess adiposity, diabetes mellitus and dyslipidaemia, as well as patients at risk of developing CVD [1]. The AHA proposes four stages of CKM, aiming at early identification of risk factors and detection of CVD in asymptomatic stages, where there is opportunity for lifestyle and pharmacological interventions that delay adverse cardiovascular outcomes [1]. CKM syndrome is regarded as a continuum, where regression is possible in early stages if adequate measures are adopted. Stage 0 reflects the absence of metabolic risk factors in individuals with normal body mass index (BMI). Stage 1 includes individuals with excess or dysfunctional adiposity, whereas stage 2 represents the development of metabolic syndrome (MetS) or the presence of significant chronic kidney disease (CKD), leading to an increased risk of developing CVD. Stage 3 includes patients with subclinical CVD or at very high risk of developing CVD, while stage 4 is characterized by progression to established CVD such as heart failure (HF), atherosclerotic cardiovascular disease (ASCVD), stroke or atrial fibrillation (AF). Given the intertwined pathogenetic mechanisms leading to CVD, as well as advances in therapeutic options with multiple CKM benefits, there is an increased need to establish a consensus for multidisciplinary care of patients across the CKM spectrum [2,3,4,5,6,7] (Figure 1).

Poor CKM health is becoming increasingly prevalent worldwide, leading to premature cardiovascular morbidity and mortality, as well as high expenditure for healthcare systems. A study by Ostrominski et al. including 11,607 patients from the National Health and Nutrition Examination Survey (NHANES) registry found that more than 25% of adults in the US had at least one cardiac, metabolic or renal condition, with growing burden over the age of 65 [8]. CKM syndrome exhibits complex physiopathological interplays, where metabolic risk factors and kidney dysfunction trigger cascading effects leading to various subtypes of CVD. As a result, forms of CVD such as coronary artery disease (CAD) and HF are highly prevalent and amongst the leading causes of death in patients with CKD and type 2 diabetes mellitus (T2DM) [9,10,11,12]. Obesity and excess adiposity are becoming increasingly prevalent in most populations and are also associated with an increased risk of CVD [13,14]. Apart from accelerated atherosclerosis due to a pro-inflammatory status, obesity induces haemodynamic changes and adverse myocardial remodelling, leading to the development of AF and HF [14,15,16]. It is therefore increasingly clear that CKM syndrome is a significant public health concern, driving early mortality and disability mainly due to cardiovascular disease. For these reasons, collaborative care and establishment of public health policies are essential in mitigating adverse outcomes related to the increasing burden of poor CKM health.

HF represents a highly prevalent form of CVD in CKM syndrome, defined as the inability of the heart to maintain adequate output without increased filling pressures. HF is associated with significantly impaired quality of life, morbidity, mortality and an important economic burden that is expected to continue to rise [17,18,19]. Current guidelines propose classification in three phenotypes, based on echocardiographic assessment of left ventricular ejection fraction (LVEF) [20,21]. The diagnosis of heart failure with reduced ejection fraction (HFrEF) (LVEF ≤ 40%) and heart failure with mildly reduced ejection fraction (HFmrEF) (LVEF between 41% and 49%) is established based on the presence of signs of HF and echocardiographic assessment of left ventricular systolic performance [20,21]. In patients with LVEF ≥ 50%, diagnosis of heart failure with preserved ejection fraction (HFpEF) requires additional evidence of structural abnormalities, increased left ventricular filling pressures or increased levels of biomarkers of myocardial dysfunction, such as elevated levels of natriuretic peptides (NPs) [20,22]. It is acknowledged that symptomatic HF is preceded by subclinical alterations to myocardial structure and function that may be reversible with timely intervention. In its most recent HF guidelines, the AHA acknowledges two subclinical stages of HF [21]. “Stage A” HF comprises patients at risk of developing HF, such as individuals with metabolic risk factors, carriers of genetic mutations or patients with familial history of cardiomyopathies, while “Stage B” HF is defined by evidence of structural changes, elevated filling pressures and persistently elevated markers of cardiac stress such as natriuretic peptides [NPs] or cardiac troponins, not explained by other conditions [21]. While current European HF guidelines address symptomatic HF, a 2023 position paper by the European Society of Cardiology (ESC) coins the concept of “heart stress”, referring to patients at risk of HF that exhibit increased levels of N-terminal pro-B-type natriuretic peptide (NT-proBNP), with or without structural and functional alterations [23]. Although different in terms of definition, the concepts provided by the AHA and the ESC create a framework that facilitates early identification of HF.

Across the CKM spectrum, there is a continuum of factors leading to alterations in myocardial structure and function, resulting in both systolic and diastolic dysfunction. In a pooled cohort analysis by Ahmad et al. including 19,249 participants at age 45 and 23,915 participants at age 55, individuals free of hypertension, diabetes or obesity had an up to 86% lower risk of incident HF and were free of HF for longer intervals than counterparts with one or more of the three risk factors [24]. Moreover, in patients with metabolic risk factors and kidney dysfunction, there is a significant overlap between ASCVD, AF and all phenotypes of HF [8,25]. HFrEF often results from CAD, whereas HFpEF appears to be more prevalent in patients with obesity, CKD, diabetes and AF [17,26,27,28]. Comorbidities and etiology of HF influence patient prognosis and outcomes, as evidenced by several HF registries. For instance, a 2017 study by Chioncel et al. assessing 9134 outpatients from the European Heart Failure Long-Term Registry found that patients with HFrEF were younger, had a higher prevalence of ASCVD and higher 1-year mortality rates compared with patients with HFpEF (8.8% versus 6.3%) [17]. Along with age and functional status, CKD was an independent predictor of poorer outcome in all HF phenotypes [17]. In the Swedish Heart Failure registry, the majority of HF patients had ASCVD irrespective of HF phenotype; the presence of ASCVD was associated with worse outcomes [29]. Both CKD and diabetes appear to be associated with worse outcomes in patients with HF [30,31]. It is therefore clear that HF is a significant challenge in patients across the CKM spectrum and that comorbidities play a key role in HF pathogenesis and disease course.

Screening and diagnostic strategies for HF have been the topic of extensive research, from development of prediction scores to identification of biomarkers and imaging tests that facilitate early diagnosis. However, to date, there is little consensus regarding optimal screening strategies that are largely applicable and accurate while also being cost-effective. Moreover, diagnosis of HF is sometimes challenging in the presence of metabolic risk factors and kidney dysfunction. This review aims to assess current evidence for early HF detection and diagnosis in the setting of CKM syndrome, from prediction scores to biomarkers, while also addressing gaps in evidence and emerging research directions in the field of biomarkers.

## 2. Methods

The authors aimed to provide an overview of current recommendations for HF screening and diagnosis in the context of CKM syndrome, as well as relevant recent research on the topic. The authors performed searches in MEDLINE/PubMEd and Web of Science using combinations of the terms “heart failure”, “systolic dysfunction”, “diastolic dysfunction”, “left ventricular systolic dysfunction”, “left ventricular diastolic dysfunction”, “preclinical”, “asymptomatic”, “subclinical” and “incident”, using Boolean operators and MESH terms to optimize search results. The search included English-language publications and considered both randomized and observational trials. Studies involving human subjects were prioritized. Reference lists were consulted in order to identify other relevant research. A narrative review was drafted, aiming to synthesize most relevant findings to date as well as future research directions.

## 3. Cardiovascular Disease in Cardiovascular–Kidney–Metabolic Syndrome: Prediction Tools

Validated prediction scores are instrumental tools for CVD prevention. Aside from estimating risk, they facilitate patient–physician communication regarding prevention goals and risk reduction strategies [32]. Their use has been most widely implemented in ASCVD, where they are essential in assessing risk and tailoring interventions. ASCVD remains the leading cause of death worldwide, as well as an important etiology of HF [33]. Although its incidence in some populations is declining, it remains a significant public health concern due to its complications and high mortality rates. Although medical, interventional and surgical treatment of CAD has progressed significantly in recent decades leading to improved outcomes, primary prevention of ASCVD remains of paramount importance. American and European CVD prevention guidelines recommend risk stratification by using validated, multivariable prediction scores that guide lifestyle and therapeutic interventions. The ESC Prevention guidelines currently endorse the use of the SCORE-2 and SCORE-2OP grids, which estimate 10-year risk of fatal and non-fatal CVD events based on age, sex, smoking status, systolic blood pressure and non-HDL cholesterol levels [32]. As a novelty to their predecessor, the SCORE grid, current scores have been extended to older patients and are calibrated for four categories of risk based on local mortality due to CVD. A similar score, the Pooled Cohort Equations, is recommended by the AHA in its most recent Primary Prevention Guidelines for patients between the ages of 40 and 79, while acknowledging the importance of recognizing other risk-enhancing factors [34]. While valuable for risk estimation in apparently healthy individuals, SCORE-2 and SCORE-2OP are not appropriate for use in patients with higher-than-average risk, such as patients with diabetes and CKD. To mitigate these limitations, the ESC developed the SCORE2-Diabetes tool to refine prediction of 10-year cardiovascular risk in patients with diabetes by adding estimated glomerular filtration rate (eGFR), age at onset of diabetes and glycated hemoglobin (HbA1c) to parameters included in the SCORE2 grid [35]. To date, there are no generally accepted biomarkers for screening of ASCVD, although some guidelines recommend testing for lipoprotein(a) (Lp(a)), apolipoprotein B (apoB) or high sensitivity C-reactive protein (hs-CRP) in select individuals for further risk stratification and more accurate prediction of CVD risk [34,36]. Lp(a) is a low-density lipoprotein cholesterol (LDL)-like molecule that has been linked with ASCVD and aortic stenosis [37]. Unlike other lipid particles, Lp(a) levels are genetically determined and appear to be relatively constant across an individual’s lifespan, although there are several factors that may increase or decrease circulating levels. While there is robust evidence of its association with various forms of CVD, Lp(a) remains underutilized in clinical practice due to a series of limitations [38]. There is significant impact of ethnicity on Lp(a) levels, as evidenced in population-based studies [39,40]. Due to heterogeneity in population-level characteristics, it is challenging to identify optimal thresholds that indicate higher CVD risk and optimal management strategies. Moreover, there are challenges pertaining to Lp(a) measurement and reporting, and there is need for further standardization of assays. Nonetheless, current European guidelines recommend once in lifetime testing of Lp(a) in order to improve risk stratification [41]. While there are no currently approved medications that reduce Lp(a) levels, there is wide opportunity for research on the topic, with several ongoing clinical trials. ApoB is present in atherogenic lipid particles and is therefore useful for directly quantifying atherogenic potential [42]. Current measurements of LDL are estimates rather than direct quantification and are subject to variability due to concomitant measurement of other molecules such as very-low-density cholesterol (VLDL-C). Moreover, LDL quantification is influenced by factors such as serum triglycerides. For this reason, apoB provides a more accurate reflection of ASCVD risk. A large meta-analysis by Sniderman et al. showed that apoB was a better predictor of cardiovascular events compared to LDL and non-high-density lipoprotein (HDL) cholesterol levels [43]. Elevated apoB levels are recognized as risk enhancers by current American and European lipid-management guidelines [44,45]. Nonetheless, there is a paucity of prospective data regarding outcomes when LDL versus apoB is used to assess risk. As apoB testing carries additional costs and provides similar information regarding risk as LDL, current guidelines endorse testing only in specific populations, such as individuals with hypertriglyceridemia [44,45]. Similarly to Lp(a), further studies investigating apoB as a biomarker of risk and potential therapeutic target will address current gaps in evidence. Although prediction scores and subsequent tailored lipid management have greatly improved ASCVD prevention, there are still unanswered questions regarding residual risk. Inflammation appears to play a key role, therefore there is an increased need to identify relevant biomarkers. Of inflammatory biomarkers, hs-CRP is perhaps the most widely available, with considerable evidence for its use. Nonetheless, its lack of specificity constitutes a major obstacle in its widespread implementation. Moreover, there is heterogeneity in results from trials investigating the accuracy of prediction in different populations, with some studies reporting only modest discrimination [46,47]. Owing to these limitations, current European prevention guidelines recommend against universal hs-CRP testing; American guidelines acknowledge its ability to refine risk but do not provide recommendations for universal testing [32,34]. More recently, two trials have assessed the use of combined testing of Lp(a), hs-CRP and LDL [48,49]. In both European and American cohorts, combined use of the three biomarkers strongly predicted long-term risk of major adverse cardiovascular events (MACE) [48,49]. Based on current evidence, there is robust evidence in favor of integrating the aforementioned biomarkers in clinical practice; target populations, frequency of testing and subsequent management strategies remain important topics for future research.

In addition to prediction scores and biomarkers, imaging techniques (most notably computed tomography (CT)) have emerged as valuable tools in refining risk assessment. Coronary artery calcium (CAC) measurement has become a valuable tool in identifying patients that might benefit from more intensive prevention strategies such as lipid-lowering therapy. The absence of coronary artery calcification is associated with a low risk of mortality, whereas increases in CAC scores predicted higher risk of mortality and major adverse cardiovascular events (MACE) [50]. Nonetheless, a CAC of 0 does not exclude the presence of non-calcified plaques and may not be entirely suitable for risk downgrading in patients with additional risk factors such as diabetes or family history of ASCVD [34]. In a cohort of 1330 patients with intermediate risk, CAC was superior to hs-CRP or family history in predicting incident ASCVD [51]. In a study evaluating combined use of computed tomography angiography (CTA) and hs-CRP, patients with diabetes exhibited significantly higher levels of hs-CRP [52]. Subjects with higher hs-CRP levels had significantly higher odds of both calcified and non-calcified plaques [52]. Due to limited availability and high expenditure associated with use of CT, a stepwise approach integrating clinical characteristics and biomarkers to identify individuals most likely to benefit from CT evaluation is a topic for future investigation.

In a recent analysis of data from 73,769 patients enrolled in the Swedish Heart Failure Registry between 2010 and 2023, ASCVD was the most prevalent etiology of HF in patients with HFrEF and HFmrEF [26]. Interestingly, trends in underlying etiology of HF changed during the observation period in HFrEF and HFmrEF patients, with a decrease in ischaemic cause and an increase in “other” causes such as genetic and acquired cardiomyopathies. While the observed changes in trends could be explained by advances in diagnosing other causes of HF, it is reasonable to hypothesize that improved prevention and management of ASCVD has led to a decrease in incident HF of ischaemic etiology. These findings highlight the importance of an integrative approach to CVD, particularly in the context of patients with metabolic risk factors and CKD.

While risk-prediction models are the cornerstone of ASCVD prevention, screening and prediction strategies for HF are less well established. Within the novel concept of CKM syndrome as a spectrum, there is a need for widely applicable prevention strategies that include risk scores accurate across diverse populations. Similarly to ASCVD, the goal is to identify patients at increased risk of developing HF, who may benefit from more frequent surveillance and interventions that delay onset of clinical HF. As a consequence, Khan et al. developed the Pooled Cohort Equations to Prevent HF (PCP-HF), which integrate easily available clinical data in a model that predicts 10-year risk of incident HF [53]. The proposed model was developed using data from ethnically diverse populations and was subsequently validated in populations from both the US and Europe, hence demonstrating its applicability in a wide range of subjects [53]. The model was further assessed in a retrospective cohort for which HF outcomes were available, showing excellent discrimination with a C-statistic of over 0.8 for White men and women, albeit with a lower discrimination for Black men and women, with a C-statistic of 0.69 for both categories [54]. Following its development, the PCP-HF equation has been included by current AHA HF guidelines in its recommendations aimed at reducing incident HF [21]. Nonetheless, guidelines acknowledge limitations of current models, particularly given the variability of individual and community-based risk factors for HF development. For this reason, there is an increased need to validate current models in a more diverse array of populations, to ensure uniformity of prediction and to avoid under- or overestimating risk.

After the introduction of the CKM framework, which highlights the importance of an integrative approach to CVD, Khan et al. developed the PREVENT equation [55]. This novel prediction model adds HF as an outcome, providing estimates of risk of total CVD (a composite of ASCVD and HF), as well as of both ASCVD and HF separately. Its use is intended for patients aged between 30 and 79 years, as the concept of CKM syndrome focuses on lifelong cardiovascular health, beginning with early adulthood [55]. Moreover, the PREVENT score can be used for patients with T2DM and CKD, as it includes kidney function (eGFR) in its prediction model in addition to traditional parameters used by the Pooled Cohort Estimates. Risk prediction is improved when HbA1c and urine albumin/creatinine ratio (uACR) are included, as both biomarkers have been shown to be predictors of cardiovascular mortality and should be routinely evaluated in patients with T2DM and CKD [56]. The PREVENT equation was subsequently validated in a large cohort of patients from NHANES for which outcomes were available [57]. In addition to providing an estimate of the risk of HF, the PREVENT model performed better than Pooled Cohort Equations in estimating 10-year CVD mortality [57]. These findings were consistent when the PREVENT equations and Pooled Cohort Estimates were applied to individuals from the prospective Multi-Ethnic Study of Atherosclerosis (MESA) cohort, as PREVENT outperformed Pooled Cohort Estimates for ASCVD prediction [58]. Although more accurate for ASCVD prediction, in the MESA cohort the PREVENT-HF equation significantly overestimated risk of incident HF, calling into question whether this novel score is appropriate for widespread clinical implementation [58]. For this reason, there is need for future research and subsequent validation of prediction scores in diverse populations. Nevertheless, the PREVENT equations represent a pivotal step in achieving the goal of an integrative approach to CKM syndrome, as they will likely contribute to early identification of patients at risk and improve shared decision-making aimed at risk reduction.

## 4. The Role of Natriuretic Peptides in the Diagnosis of Heart Failure

While prediction scores are valuable for screening large populations and can be applied in a wide array of clinical settings that include primary care, a significant proportion of patients at risk might benefit from additional tests that facilitate early diagnosis of HF. For this reason, there is an increased need to develop cost-effective and reasonably accurate tools that aid in identification of subclinical cardiac dysfunction. In a recent statement, the AHA advocates for a stepwise approach to primary prevention of HF, from prediction scores that are widely applicable, to identifying patient-level risk enhancers and proceeding to additional testing in patients at greatest risk [59]. Risk refinement can be achieved by using a biomarker-based approach, with most evidence supporting the use of NPs such as NT-proBNP and B-type natriuretic peptide (BNP). Although not a diagnostic criterion per se, NPs are also highly useful for diagnosing overt HF, as symptoms and signs are often non-specific. This is especially true for HFpEF, a phenotype of HF highly prevalent in patients with metabolic risk factors and CKD, where elevated NPs levels support diagnosis [22]. Despite extensive data supporting the use of NPs to detect preclinical and clinical HF, there are considerable factors that influence circulating levels such as age, kidney function, obesity and the presence of atrial arrhythmias such as AF, all of which are commonly encountered across the CKM spectrum. As a consequence of limitations in the use of NPs, there is great opportunity for research in the field of biomarkers of HF. Data from observational studies in a wide array of clinical settings has yielded conflicting results, therefore no additional biomarkers are endorsed by current guidelines. Nonetheless, novel biomarkers could provide additional insight into mechanisms leading to development of HF, such as inflammation, myocardial remodelling, fibrosis and endothelial dysfunction.

### 4.1. Natriuretic Peptides: An Overview

NPs are the most widely used and researched biomarkers of HF. NPs are secreted by myocytes in response to myocardial stretching due to volume overload and increased filling pressures, promoting diuresis and natriuresis [60]. From a biochemical standpoint, a prehormone comprising 134 amino acids, preproBNP, is enzymatically cleaved to proBNP, which is further cleaved to the active compound BNP and the biologically inactive compound NT-proBNP, both of which can be determined in serum samples [61]. BNP exerts its effects mainly by binding to the natriuretic peptide receptor-A (NPR-A) receptor, which induces cyclic guanosine monophospate (cGMP) synthesis leading to natriuresis, diuresis and vasodilation; effects from binding to the natriuretic peptide receptor-B (NPR-B) receptor are less clear [61]. Clearance mechanisms differ for BNP and NT-proBNP. BNP degradation occurs by binding to the natriuretic peptide receptor-C (NPR-C) receptor and cleavage by the ubiquitous enzyme neprilysin [62,63]. NT-proBNP is mainly excreted by the kidneys and has a longer half-life compared to BNP, leading to higher serum concentrations [64].

Assessment of NPs is useful in a variety of clinical scenarios. Although not mandatory to establish the diagnosis, current guidelines encourage NPs testing in all patients with suspected chronic HF, for both diagnostic and risk-stratification purposes [20,21]. In patients with suspected chronic HF, the ESC HF guideline uses values of <35 pg/mL for BNP and <125 pg/mL for NT-proBNP as a cut-off at which a diagnosis of HF is deemed unlikely [20]. In acute settings, NPs are valuable tools when evaluating patients presenting with dyspnea, as differential diagnosis includes a wide array of non-cardiac causes. Several landmark studies such as ‘Breathing Not Properly’ and ‘Pro-BNP investigation of dyspnea in the emergency department’ (PRIDE) evaluated the utility of NPs testing for diagnosing acute HF [65,66]. After validation in various cohorts, cut-off values of BNP < 100 pg/mL or NT-proBNP < 300 pg/mL were deemed suitable to confidently exclude a diagnosis of acute HF, with further recommendation for age-adjusted “rule-in” values. As mentioned, there are several factors that may influence circulating NPs levels, although the clinical relevance of this phenomenon is not entirely elucidated. In the setting of CKM syndrome, this is a topic of significant interest, as levels are increased in patients with decreased kidney function or AF and are lower in patients with higher BMI. Currently, specific thresholds for both BNP and NT-proBNP are recommended in patients with AF as part of the diagnostic algorithm of HFpEF [22]. While no formal adjustments are recommended for age, BMI or eGFR by current European HF guidelines, it is worth mentioning that a 2023 ESC position paper includes additional information on how factors that significantly affect NT-proBNP levels should be taken into consideration [23]. Moreover, for both acute and chronic settings, after age adjustments are taken into consideration, there are so-called “gray areas” of NPs levels, in which HF is unlikely but cannot be confidently excluded; patients falling into this category usually require either follow-up or additional investigations in order to establish a definite diagnosis.

NPs have also been extensively studied for their role in predicting development of HF and for their use for screening purposes. Early research on the topic yielded conflicting results, possibly due to considerable heterogeneity in study population characteristics and inclusion or exclusion criteria. In 2002, Vasan et al. conducted a study assessing diagnostic performance of BNP and N-terminal atrial natriuretic peptide (NT-ANP) in detecting asymptomatic left ventricular hypertrophy and left ventricular systolic dysfunction (defined as LVEF ≤ 50%) in a cohort of participants from the Framingham Offspring Study, excluding patients with documented HF but including patients with risk factors such as ASCVD or hypertension [67]. Area under the curve (AUC) derived from Receiver Operating Characteristics (ROC) curves was low for both natriuretic peptides (below 0.75), with poorer performance in women, suggesting that population-wide NPs screening was of limited usefulness in detecting subclinical systolic cardiac dysfunction [67]. By contrast, a subsequent study by Ng et al. screening 1360 patients found excellent AUC for BNP in identifying significant yet asymptomatic left ventricular systolic dysfunction, especially when used in conjunction with other data such as major electrocardiographic abnormalities and history of CAD [68]. Both studies had limitations, such as low overall prevalence of left ventricular systolic dysfunction; there were also differences in methods by which systolic dysfunction was defined and quantified, therefore results between studies may not be comparable. McGrady et al. evaluated NT-proBNP levels in 3550 patients aged over 60 who had at least one risk factor for developing incident HF, such as CKD, AF, CAD or T2DM [69]. Patients with NT-proBNP of over 254 pg/mL (the fifth quintile) subsequently underwent echocardiography with focused assessment on diastolic dysfunction, which is acknowledged as a cornerstone in the development HFpEF. A total of 25% of patients with elevated NT-proBNP exhibited moderate or severe diastolic dysfunction. As expected, median plasma NT-proBNP significantly increased with severity of diastolic dysfunction, from 355 pg/mL in patients with no dysfunction to 448 pg/mL in moderate or severe diastolic dysfunction. While this study provided valuable insight regarding detection of significant asymptomatic diastolic dysfunction using an NP-based screening protocol, it is worth noting that the threshold used for referral to echocardiography was higher than the current threshold recommended by the ESC consensus for patients aged below 75. Nonetheless, results from these early studies proved that NP-based screening could be useful in detecting patients with asymptomatic alterations of myocardial function. Although most results favored use of NPs in assessing risk, conflicting results of observational studies regarding NP-based screening led to the design of randomized studies to address gaps in evidence. In 2013, Ledwidge et al. conducted the first randomized clinical trial assessing the role of BNP in screening for HF in patients at risk [70]. The study included 1374 patients with metabolic risk factors (hypertension, diabetes, hypercholesterolemia) or ASCVD, with no evidence of HF at enrollment, and randomly assigned them to either receive BNP testing or usual care. A cut-off level for BNP of >50 pg/mL was used for referral to evaluation by a cardiologist and subsequent follow-up, as well as adjustment of preventive interventions [70]. The primary endpoint was a composite of left ventricular dysfunction (both systolic and diastolic) as well as incident HF. Patients in the intervention group had a statistically significant (*p* = 0.003) lower odds ratio (OR) of reaching the primary endpoint compared to the control group, as well a significantly lower rate of major adverse cardiac events (MACE) (OR, 0.69; 95% CI, 0.49–0.98; *p* = 0.04) [70]. Although the study had limitations such as patient heterogeneity and exclusion of outpatient diagnosis of new-onset HF and MACE, it provided valuable information regarding the utility of BNP-based screening and tailoring of intervention in patients deemed to be at risk of developing HF. In a similar fashion, the PONTIAC trial evaluated NT-proBNP-guided up-titration of cardioprotective medication in patients with T2DM free of cardiac disease [71]. Patients in the intervention group had significantly lower rates of reaching the primary endpoint of cardiac hospitalization or death (HR: 0.351; 95% CI 0.127 to 0.975, *p* = 0.044), as well as for secondary endpoint such as HF hospitalizations [71]. Given the evidence provided by observational and randomized trials, several current guidelines formally recommend the use of NPs for screening purposes. AHA HF guidelines include a class IIA recommendation for the use of NPs in patients at risk of developing HF (stage A HF), while the American Diabetes Association (ADA) currently recommends considering screening for asymptomatic HF in patients with diabetes by using NPs and subsequent referral for further investigation in case of abnormal results [72]. The ESC position paper regarding early diagnosis of HF by using NT-proBNP uses a threshold of ≤50 pg/mL at which subclinical heart stress is unlikely, recommending repeat testing once a year [23]. Patients with results in the “gray area” may warrant additional testing at shorter intervals of time, whereas patients with “rule-in” results might benefit from early echocardiographic evaluation, as well as more aggressive risk factor control. As proposed by the AHA statement regarding primary prevention of HF, NPs measurement could be used in conjunction with risk-prediction models to improve their accuracy. For instance, a 2012 study by Agarwal et al. found that NT-proBNP demonstrated the ability to significantly improve prediction of incident HF when added to a risk model in patients from the Atherosclerosis Risk in Communities (ARIC) cohort [73]. The ARIC HF risk model displayed an AUC of 0.773 (95% CI, 0.753–0.787), which improved significantly to 0.805 (95% CI, 0.792–0.820) when NT-proBNP was included, with a net reclassification improvement (NRI) of 13% (95% CI, 10.2–19.9%) [73]. While it remains unclear if widespread NP-based screening is accurate as well as cost-effective, it is evident that it provides valuable information in patients with CKM syndrome. Nonetheless, there are limitations to this approach, mainly due to the aforementioned possible confounders.

#### 4.1.1. Natriuretic Peptides and Obesity

Excess adiposity appears to be associated with lower circulating levels of NPs [74,75,76]. Mechanisms through which this phenomenon occurs are incompletely elucidated, given the different clearance pathways for BNP and NT-proBNP [64,77]. Possible explanations include reduced release of NPs, increased renal clearance as a result of higher glomerular filtration rate and the presence of NPR-C receptors on the surface of adipose tissue [78,79]. BMI, as well as disposition of excess adiposity (particularly visceral adipose tissue, as opposed to subcutaneous adipose tissue), have been reported to be associated with lower circulating NT-proBNP levels [75]. As obesity is a highly important feature of CKM syndrome, the inverse relationship between excess adiposity and NPs constitutes a significant challenge. Although virtually all current guidelines advise caution when interpreting NPs levels in individuals with excess weight, the extent of the clinical relevance of this inverse association is yet unclear and therefore there are no formal adjustments of NPs threshold with respect to BMI.

In a large cohort of Dutch patients free of HF at baseline, Suthahar et al. investigated the complex interactions between anthropometric parameters, sex, age and NT-proBNP levels [80]. The study found higher baseline NT-proBNP levels in females. In contrast to findings from similar studies, men displayed higher NT-proBNP levels with increasing BMI and waist circumference (*p* < 0.05). In multivariate analysis, waist circumference displayed a weak (Sβ = −0.076) but statistically significant (*p* = 0.014) association with lower levels of NT-proBNP, but only in females [80]. Findings from this study suggest that variables such as sex and age are possible confounders when evaluating the relationship between anthropometric parameters and circulating NPs levels. An analysis by Ndumele et al. of 12,230 patients in the ARIC cohort evaluated the utility of NT-proBNP testing in conjunction with the ARIC HF score in predicting incident HF, comparing findings between patients with and without obesity [81]. The study found an overall inverse association between BMI and circulating NT-proBNP, consistent with most of the previous reports [81]. Importantly, NT-proBNP retained its ability to predict incident HF even in patients with obesity, as greater excess weight was associated with an increased rate of incident HF (16.8 per 1000 person-years incident rate for severe obesity) [81]. NT-proBNP levels predicted incident HF even after adjustment to parameters included in the ARIC HF risk model and increases in NT-proBNP levels from specified cut-off points predicted greater absolute risk of incident HF in individuals with obesity and severe obesity [81]. Although this study did not include other anthropometric parameters and defined obesity solely based on BMI, its findings support the use of NT-proBNP as an instrument of risk stratification in patients with excess weight. Contrasting results between studies are possibly a result of inherently different population subsets, as well as heterogeneous study methods and parameters used to define and quantify obesity.

Appropriate cut-offs for NPs in overt HF in patients with obesity are still a matter of debate. A significant proportion of patients with concomitant obesity and HF display NPs values below current “rule out” thresholds; this is relevant particularly in the HFpEF phenotype, which is commonly encountered in patients with obesity and for which NP testing is an important diagnostic tool. Early studies reported the inverse relationship between NPs and BMI in both acute and chronic HF. A substudy of the PRIDE study including patients with acute HF showed lower levels of circulating NPs in patients with obesity (BMI ≥ 30 kg/m^2^) and overweight (BMI 25–29.9 kg/m^2^) compared to patients with a BMI ≤ 25 kg/m^2^ (*p* < 0.001 for both), even after adjustment for covariates [82]. At a cut-off of 900 pg/mL, NT-proBNP had a sensitivity of 87% in patients with a BMI > 25 kg/m^2^ [82]. NT-proBNP appeared to be affected by BMI to a lesser extent than BNP, possibly as a result of different clearance mechanisms, suggesting that NT-proBNP could be superior to BNP when assessed in individuals with excess weight [82]. An observational study by Vaishnav et al. including 89 patients with obesity and HFpEF hospitalized for AHF found that individuals with most severe obesity (defined as a BMI ≥ 40.0 kg/m^2^) had significantly lower admission NT-proBNP levels of 670.5 pg/mL (interquartile range (IQR): 128–1268) compared to patients without obesity (BMI < 30.0 kg/m^2^), in which median NT-proBNP was 2607 pg/mL (IQR: 2112–5703), or patients with obesity (BMI 30.0–39.9 kg/m^2^) in which median NT-proBNP was 1725 pg/mL (IQR: 889–3900) [83]. Interestingly, 1-year outcomes were in line with the so-called “obesity paradox” in HF, as patients in the highest BMI category had significantly better survival [83,84,85]. An important finding from this study was that 12 out of 89 of the included patients exhibited NT-proBNP levels below to currently recommended cut-off for acute HF, providing further evidence that BMI-adjusted cut-offs might be required [83]. Verbrugge et al. conducted a study assessing 581 patients undergoing diagnostic work-up for dyspnea, of which 420 patients were diagnosed with HFpEF based on invasive cardiopulmonary exercise testing [86]. The study found that 157 of the patients diagnosed with HFpEF had “normal” NT-proBNP levels (<125 pg/mL) [86]. Mean BMI in patients with normal NT-proBNP HFpEF was 35.3 kg/m^2^, higher than that of counterparts with elevated NT-proBNP HFpEF [86]. Patients with elevated NPs displayed more severe structural and functional abnormalities and lower cardiac output reserve with exercise compared with patients with normal NT-proBNP (85% predicted, IQR: 59–109%; *p* < 0.001) [86]. Moreover, patients with normal NT-proBNP HFpEF were less likely to reach the primary endpoint compared to counterparts with high NT-proBNP [86]. The findings of this study were two-fold; on the one hand, it demonstrated that the current guideline-recommended NT-proBNP cut-off for chronic HF might not be appropriate in patients with excess weight; on the other hand, it presented evidence that lower NT-proBNP is possibly associated with less severe HF and better outcomes, confirming its value when evaluating HF patients irrespective of weight [86]. A similar study by Reddy et al. found higher prevalence of obesity, AF and CKD in patients diagnosed with HFpEF by invasive testing [87]. An NT-proBNP cut-off of <125 pg/mL displayed a low sensitivity of 67% (95% CI, 58–77%) in patients with BMI ≥ 35 kg/m^2^; lowering the threshold to <50 pg/mL provided improved sensitivity of 86% (95% CI, 79–93%) [87].

Based on findings from these studies, it is increasingly clear that interpretation of NPs levels in patients with obesity warrants careful consideration, whether they be used for screening or diagnostic purposes (Table 1). There is wide opportunity for further research on the topic in order to elucidate mechanisms by which circulating NPs levels are lower in individuals with excess weight, as well as clinical implications. Well-designed, prospective studies may provide adjustment of current thresholds so as to reduce underrecognition of preclinical and clinical HF in patients with obesity.

#### 4.1.2. Natriuretic Peptides and CKD

In the setting of CKM syndrome, complex interactions between heart and kidneys and the frequent association of HF and CKD pose unique challenges. Patients with HF often exhibit renal dysfunction and CKD confers worse prognosis in HF patients [85,88,89]. Owing to their participation in clearance of NPs (mainly NT-proBNP), kidney dysfunction is associated with an increase in serum NPs levels that impacts their use in clinical practice. While BNP is mainly degraded by binding to the NPR-C receptor and by circulating proteases, NT-proBNP is thought to be mainly excreted by the kidneys. Palmer et al. obtained blood samples from different arterial and venous sites in 120 patients undergoing cardiac catheterization, finding that between 55 and 65% of NT-proBNP is cleared by the kidneys [90]. The study also confirmed an inverse relationship between eGFR and arterial levels of NT-proBNP [90]. An important finding of this study was that fractional tissue extraction of NT-proBNP at kidney level was unaffected by eGFR, suggesting that elevated NT-proBNP levels in this setting are likely a result of both increased secretion by cardiac tissue and diminished clearance [90]. Perhaps more surprisingly given the mechanisms by which BNP is cleared, a study by Tsutamoto et al. found that BNP levels were also inversely associated with eGFR irrespective of severity of HF [91].

Although impaired kidney function increases circulating troponin levels as well as NT-proBNP levels, a study conducted by Bansal et al. in 3483 patients from the Chronic Renal Insufficiency Cohort (CRIC) with no prior diagnosis of HF found that both NT-proBNP and cardiac troponin retain their ability to predict incident HF in patients with CKD [92]. NT-proBNP was independently associated with risk of incident HF, as well as of both HFrEF and HFpEF, with a hazard ratio of 3.01 (95% CI, 2.37–3.84) and 1.64 (95% CI, 1.32–2.04), respectively [92]. Subsequently, Bansal et al. evaluated the value of dynamic monitoring of serum biomarkers in a cohort of participants enrolled in the CRIC study free of HF and AF at baseline [93]. On average, NT-proBNP levels increased by 50 pg/mL between measurements [93]. The highest quartile of dynamic change for NT-proBNP was associated with an unadjusted hazard ratio of 2.61 (95% CI 1.69, 4.05) of incident HF; moreover, dynamic changes in NT-proBNP predicted incident AF [93]. Henceforth, while it is true that levels of circulating NT-proBNP are influenced by kidney function, this likely reflects subclinical pathogenic processes that ultimately lead to development of HF. While NT-proBNP is more frequently used in clinical practice due to its longer plasma half-life, Hayashida et al. investigated the use of BNP in a cohort of patients with significant CKD (median eGFR 30.3 mL/min/1.73 m^2^) [94]. After adjustment for potential confounders, BNP independently predicted risk of fatal and non-fatal cardiovascular events, particularly in patients with preexisting CVD [94]. Current HF guidelines do not make formal recommendations for eGFR-based adjustments of cut-offs [20,21]. The European practice statement for clinical use of NT-proBNP advises against adjusting cut-off levels of NT-proBNP in emergency setting to kidney function but recommends considering adjustments (increasing the threshold) in outpatient settings [23]. Despite robust evidence in favor of NPs use in patients with kidney dysfunction, there is opportunity for research evaluating biomarkers that are affected to a lesser extent by CKD.

## 5. Additional Biomarkers

NPs and troponins are the only biomarkers with sufficient evidence for screening and diagnosis of HF [20,21,72,95]. Nonetheless, several other biomarkers are of interest, particularly in the setting of CKM-associated HF. As mentioned previously, numerous molecules that could serve as biomarkers and as potential therapeutic targets have been investigated, but to date none are formally included in CVD prevention and HF guidelines. This is likely a result of significant heterogeneity among observational studies, from characteristics inherent to the investigated populations, to ways in which outcomes are defined and reported. Nonetheless, we decided to briefly review evidence pertaining to the most promising biomarkers to date.

### 5.1. Biomarkers of Inflammation

Advances in research of underlying mechanisms of HF have highlighted the pivotal role of inflammation, particularly in HFpEF [96]. This association is of significant relevance in the setting of CKM-associated HF, as dysfunctional and excessive adipose tissue releases numerous pro-inflammatory cytokines, leading to endothelial dysfunction, interstitial fibrosis and myocardial stiffening [97]. Inflammation is also associated with accelerated atherosclerosis, increasing the likelihood of developing HF as a consequence of CAD. Hs-CRP and C-reactive protein (CRP) are perhaps the most widely used biomarkers of inflammation, reflecting the activation of the interleukin-6 (Il-6) pathway. Persistently elevated hs-CRP levels have been shown to be associated with a heightened risk of cardiovascular mortality and MACE [98]. Given the link between inflammation, ASCVD and HF, several cohort-based studies have evaluated the utility of hs-CRP and CRP in predicting cardiovascular outcomes. A substudy of the ARIC cohort showed that elevated levels of hs-CRP were associated with an increased risk of meeting the primary endpoint of ASCVD events (myocardial infarction, stroke or death related to an ASCVD event), as well as the secondary endpoint of incident HF, irrespective of lipid profile and risk estimates based on the Pooled Cohort Equations [99]. In the MESA cohort, hs-CRP independently predicted incident HF in patients undergoing treatment with statins, while prediction of ASCVD events was more modest [100]. Kalogeropoulos et al. found that in elder adults (aged 70–79), markers of inflammation such as IL-6, tumor necrosis factor α (TNF-α) and hs-CRP were associated with an increased risk of developing incident HF, and in particular with HFpEF, although hs-CRP association was weaker [101]. IL-6 levels more strongly predicted HF of non-ischaemic cause, whereas TNF-α predicted HF in patients with ASCVD [101]. In a subset of participants from the Prevention of Renal and Vascular End-Stage Disease (PREVEND) study, IL-6 was associated with an increased likelihood of several metabolic risk factors (increased BMI, hypertension, diabetes), as well as with increasing age [102]. In line with previous reports, in this cohort IL-6 was consistently associated with incident HFpEF to various extents, depending on models employed for multivariate analysis; there was no association with incident HFrEF [102]. Conflicting results regarding the value of inflammatory biomarkers in predicting HF could partially be explained by population heterogeneity, low specificity, numerous possible confounders, as well as other inflammatory pathways resulting in cardiac damage. Nonetheless, research into the field is of paramount importance, as inflammation is a cornerstone of CVD related to metabolic risk factors and may prove to be an important therapeutic target.

### 5.2. sST2

Soluble suppression of tumorigenicity 2 (sST2) is the soluble form of the receptor for interleukin-33 (IL-33) [103]. The IL-33/ST2 complex is a marker of myocardial stress and appears to exert cardioprotective effects, such as inhibition of apoptosis and reduction in myocardial fibrosis [103]. sST2 binds IL-33, counteracting its cardioprotective effects. Early evidence based on animal models showed that IL-33 and sST2 are expressed by cardiomyocites and fibroblasts as a response to different forms of stress [104]. Due to its expression in a wide array of tissues, use of sST2 for diagnosis of HF is limited. The most robust data favouring utilization of sST2 in clinical practice pertains to its ability to predict worse outcomes. In acutely decompensated HF, Lassus et al. showed that while mid-regional pro-adrenomedullin (MR-proADM) performed slightly better than sST2 in improving AUC for 30-day mortality as an addition to a clinical model, sST2 was superior with regard to 1-year mortality [105]. A meta-analysis by Aimo et al. found that in 4835 patients with acute HF, both admission and discharge sST2 predicted mortality and rehospitalization [106]. Similar findings were reported in a meta-analysis of outpatient HF, where sST2 was found to be an independent predictor of cardiovascular mortality (HR 1.79, 95% CI: 1.22 to 2.63) and all cause death (1.75, 95% CI: 1.37 to 2.22) [107]. In the setting of CKM, an important argument in favour of integrating sST2 is that its levels appear to be influenced by eGFR to a lesser extent than NPs and cardiac troponins, maintaining its prognostic value regarding all-cause mortality irrespective of kidney function [108]. Moreover, in more advanced HF (defined by worse functional status according to the New York Heart Association [NYHA] scale), sST2 levels remains constant, irrespective of eGFR [108]. The role of sST2 in screening and predicting incident HF is less well established. In a large study including 22,756 patients with from four cohorts (Framingham Heart Study, PREVEND, MESA and Cardiovascular HealthStudy), sST2 levels were not associated with incident HF in either men or women, as opposed to NT-proBNP, cardiac troponin, uACR and CRP [109]. In a cohort of elderly patients (mean age 72.7 years), sST2 levels were higher in patients with metabolic risk factors and kidney dysfunction; despite this, sST2 did not provide improved prediction compared to clinical variables and conventional biomarkers [110]. By contrast, in the CRIC, sST2 was less affected by declining eGFR than other biomarkers; dynamic increase in sST2 levels at 2 years was associated with incident HF [93]. Although sST2 therefore appears useful to predict HF in CKD patients, it is worth mentioning that this study excluded patients with end-stage renal disease [ESRD], which represent a particularly vulnerable category in which most approved screening strategies are not applicable or reliable [93]. Based on findings from these studies, it appears that sST2 is more suitable to predict outcomes in clinical HF; nevertheless, due to its variation to a lesser extent with declines in eGFR, it could become a valuable tool in the landscape of CKM syndrome.

### 5.3. VCAM-1/ICAM-1

Vascular cellular adhesion molecule-1 (VCAM-1) and intracellular adhesion molecule-1 (ICAM-1) are ubiquitous proteins belonging to the immunoglobulin superfamily. As markers of endothelial activation, VCAM-1 and ICAM-1 play a role in the development of different forms of CVD by promoting leucocyte adhesion and inflammation [111]. In an animal model in which myocardial strain was induced by Angiotensin II, VCAM-1 appeared to mediate adverse cardiac remodelling; blockade of VCAM-1 was associated with improvement in cardiac remodelling and a decrease in pro-inflammatory cytokines [112]. Based on these properties, VCAM-1 and ICAM-1 have been studied for screening, diagnostic and prognostic purposes in several forms of CVD. Early evidence from 792 participants in the ARIC cohort found no difference in circulating VCAM-1 levels between patients with ASCVD and controls, but ICAM-1 independently predicted incident CAD and carotid artery atherosclerosis [113]. In a population of 1246 patients with documented CAD (defined as angiographically proven stenosis > 30% in a coronary artery), higher levels of VCAM-1 were associated with increased risk of ASCVD mortality, independently of other predictors [114]. Apart from being a key mediator in development and progression of ASCVD, endothelial dysfunction plays an important role in development of HF. Patel et al. found that in 2297 participants from the MESA cohort, higher VCAM-1 levels were associated with incident HF [115]. VCAM-1 levels were higher in patients with metabolic risk factors, lower eGFR and increased uACR; moreover, after multivariate analysis, VCAM-1 was found to be associated with incident HFpEF but not HFrEF [115]. In addition, in a cohort of young adults in which levels of adhesion molecules were dynamically assessed, there was an association with lower global longitudinal strain levels (GLS), which is indicative of subclinical systolic dysfunction [116]. Adhesion molecules have also been studied in relation to development of AF; in a cohort of 909 participants in which levels of 13 inflammatory markers were assessed, VCAM-1 was the only one independently associated with incident AF, possibly by reflecting underlying adverse remodelling [117]. Mathew et al. found that higher VCAM-1 and ICAM-1 levels were associated with increased atrial volume and decreased peak left atrial longitudinal strain, assessed by cardiac magnetic resonance imaging (CMR); in contrast to previous findings, there was no association between adhesion molecules and left ventricular structural and functional abnormalities, including the presence of interstitial myocardial fibrosis [118]. Left atrial dysfunction is linked to the development of HFpEF and AF, therefore adhesion molecules could provide further insight and a potential therapeutic target in this particular phenotype of CVD. There is also a potential role of adhesion molecules in predicting outcomes of patients with HF. VCAM-1, but not ICAM-1, was associated with poorer outcomes in a substudy of the landmark DAPA-HF trial evaluating the role of dapagliflozin in patients with HFrEF [119]. Based on these findings, adhesion molecules appear to be promising instruments in assessing pathways leading to development of CVD. Limitations of their use include their lack of cardiac specificity; therefore, more experimental and real-world data is needed to better define their role.

### 5.4. GDF-15

Growth-differentiation factor-15 (GDF-15), a member of the transforming growth factor- β superfamily, appears to exert cardioprotective effects such as limiting cell apoptosis as a consequence of myocardial ischaemia [120]. Early research focused on its use in relation to CAD. In the Dallas Heart Study, Rohatgi et al. found that increased levels of GDF-15 were independent predictors of an increased coronary artery calcium (CAC) score, as well as of all-cause and cardiovascular mortality [121]. In an individual patient meta-analysis assessing data from 53,486 patients, elevated levels of GDF-15 served as an independent predictor of HF hospitalization and mortality in patients with ASCVD, particularly in patients with cardiometabolic risk factors such as diabetes and CKD [122]. Findings from this large study support its potential use as an instrument to identify vulnerable ASCVD patients that may warrant more intense surveillance and measures of secondary prevention [122]. Similarly to sST2, GDF-15 independently predicts worse outcomes in patients with HF [123]. In community-based settings, there is evidence that GDF-15 and sST2 are independent predictors of incident HF [124]. Xanthakis et al. hypothesized that integrating both sST2 and GDF-15 into a multimarker score could provide incremental value in detecting precursors of HF such as left ventricular hypertrophy (LVH) or left ventricular systolic dysfunction (LVSD) [125]. GDF-15 was independently associated with LVSD, whereas sST2 was not associated with either LVSD or LVH; addition of sST2 and GDF-15 did not significantly improve AUC for detection of subclinical cardiac dysfunction compared to models based on risk factors and BNP [125]. Nonetheless, in a more recent analysis of data from a larger patient population subset included in the ARIC cohort, GDF-15 was associated with incident HF in patients without diabetes (HR 1.64, 95% CI 1.41, 1.91) and more strongly in patients with diabetes (HR 1.72, 95% CI 1.32, 2.23), independently of cardiac troponin and NT-proBNP [126]. Based on findings from these studies, GDF-15 is a promising biomarker within the framework of CKM syndrome; prospective studies could identify circumstances in which it would be most useful in clinical practice.

The aforementioned biomarkers offer promise across the CKM spectrum, despite none of them being formally endorsed by current prevention and treatment guidelines (Table 2). Given their ability to offer insight into pathogenic mechanisms of CVD, there is wide opportunity for translational and clinical research that will assess their role in the management of patients.

## 6. Emerging Research Directions: Metabolic Biomarkers and Gut Microbiota

The development of metabolic syndrome (MetS) is the cornerstone of CKM-related CVD and is associated with a significant risk of developing incident HF [127,128]. Moreover, in patients with established HF, MetS appears to be associated with worse NYHA class and is linked with an increased risk of developing composite cardiovascular outcomes, although current evidence is conflicting [129,130]. There is growing interest in identifying biomarkers that reflect mechanisms through which MetS leads to HF and how the presence of MetS and its components influence outcomes. A study investigating a wide array of protein biomarkers in patients with HF found that patients with HF and MetS exhibited increased levels of adipose-tissue-derived biomarkers such as leptin, as well as molecules implicated in pro-inflammatory pathways such as interleukin-1 receptor antagonist or tumor necrosis factor receptor superfamily member 11a [130]. Leptin, a hormone derived from adipose tissue, plays a key role in regulating metabolism and appetite. Leptin resistance occurs frequently in patients with obesity, through mechanisms such as reduced expression of leptin receptors [131]. Despite robust evidence implicating leptin in progression of MetS, there is conflicting evidence regarding the role of leptin in predicting cardiovascular outcomes. Sattar et al. found that leptin levels were associated with inflammation and components of MetS such as hypertension and lipid levels, but not with incident CAD after adjustment for BMI [132]. In a population of 4080 men, higher levels of circulating leptin were associated with incident HF but not with an increased risk of MACE [133]. In a more diverse population of patients from the MESA cohort, leptin levels were not associated with MACE [134]. Additional adipokines, such as adiponectin and resistin have been linked with increased odds of cardiovascular mortality [135,136]. Based on these findings, there is a need for prospective studies to elucidate ways in which adipokines could be integrated as biomarkers of CVD risk in clinical practice. Along with the pro-inflammatory activity of excess adipose tissue, insulin resistance is a key mediator for CVD development in patients with MetS. Currently, a practical way of assessing insulin resistance is the homeostatic model assessment of insulin resistance (HOMA-IR). In a population of 22,681 patients from community-based cohorts, HOMA-IR was associated with incident HFpEF and mediated the relationship between obesity and HFpEF in non-diabetic patients [27]. Additional biomarkers of insulin resistance, such as the Triglyceride-Glucose Index (TyG), the Triglyceride-Glucose Index and Body Mass Index Product (TyG-BMI) and TyG-waist circumference Index (TyG-WC) have also been shown to predict incident HF [137]. Moreover, biomarker surrogates of insulin resistance appear to predict worse outcomes in patients with established HF [138,139]. Nonetheless, a recent analysis of 276 patients with HFpEF showed no association between HOMA-IR and cardiac remodeling or pulmonary capillary wedge pressure, which were instead associated with increased adiposity [140]. Given these conflicting findings, there is wide opportunity for clinical trials investigating biomarkers of insulin resistance and their role in furthering the current understanding of CVD progression in patients across the CKM spectrum.

There is mounting evidence that gut microbiota plays a key role in modulating pathophysiological mechanisms leading to the development and progression of CKM syndrome, such as inflammation, endothelial dysfunction and insulin resistance [141,142]. While it is difficult to standardize gut microbioma compositions, the most widely present bacteria belong to the phyla Bacteroidetes, Firmicutes, Actinobacteria and Proteobacteria. Imbalances in proportions between different phyla have been linked to various forms of CVD and outcomes. Certain compounds resulting from bacterial metabolism, such as), exert anti-inflammatory effects, whereas other compounds such as trimethylamine N-oxide (TMAO) are linked to an increased risk of adverse cardiovascular events [143,144]. In a case–control study of patients with acute ischaemic stroke or transient ischaemic attack, patients with cerebrovascular events showed significantly different gut microbiota composition compared to controls, with increased levels of Proteobacteria and reduced levels of Bacteroides, Prevotella, and Faecalibacterium [145]. Contrary to previously reported data implicating TMAO in MACE, this study found lower levels of TMAO in stroke patients. Karlsson et al. performed analysis of the gut metagenome in 12 patients with acute cerebrovascular events and 13 healthy controls, finding an increase in genes associated with peptidoglycan synthesis in patients [146]. By contrast, healthy subjects exhibited a gut microbiota profile which favoured synthesis of products with antioxidant properties [146]. Additionally, a study by Jie et al. comparing patients with stable angina, unstable angina, or acute myocardial infarction and healthy controls found significant differences in gut microbiota composition, with a reduction in Bacteroides and Prevotella and enrichment in Streptococcus and Escherichia in patients with ASCVD [147]. The gut microbiota profile of ASCVD patients resulted in lower potential for synthesis of protective SCFAs such as butyrate and propionate, with increased synthesis of enzymes involved in synthesis of TMAO [147]. In patients with HF, Luedde et al. found lower bacterial diversity compared to controls, with depletion of the genera Blautia and Collinsella and the genus Faecalibacterium, all of which are involved in synthesis of anti-inflammatory compounds [148]. It is known that diet significantly influences gut microbiota. Recently, a novel score was proposed to quantify a diet with beneficial effect on gut microbiota—the dietary index for gut microbiota (DI-GM), with points awarded for intake of beneficial nutrients and for lower intake of harmful elements [149]. In a subsequent study of patients from the NHANES cohort, a lower DI-GM was associated with the onset of CKM syndrome [150]. There is evidence that Mediterranean-type diets are associated with lower risk of CVD, possibly through favourable modulation of gut microbiota [151,152,153]. Based on these findings, it is clear that there is wide opportunity for research into the role of gut microbiota across the CKM spectrum, due to its ability to serve as a potential biomarker as well as a therapeutic target.

## 7. Conclusions

The introduction of the concept of CKM syndrome has led to a change in paradigm when approaching CVD. Given the high, growing burden of poor CKM health, prevention and early diagnosis of CVD is of paramount importance. Despite extensive research into screening and diagnostic tools, there are still important gaps in evidence when approaching CVD in the context of CKM syndrome. Novel prediction equations provide important guidance, but there is considerable heterogeneity with regard to CVD progression among different populations and with respect to individual risk factors. For this reason, integrating biomarker-based strategies into CKM risk assessment may represent a key step toward more personalized and preventive cardiovascular care. Although results from population-based observational studies of novel biomarkers have yielded conflicting results, several have been proven to provide valuable insight into various mechanisms involved in CVD development and progression in patients with cardiometabolic risk factors. Moreover, biomarkers can also serve to identify potential therapeutic targets. Although techniques such as speckle-tracking echocardiography and cardiac magnetic resonance can detect alterations to myocardial structure and function before overt symptoms develop, their use for prevention and screening purposes is largely limited due to lower availability and high costs. This review, while not exhaustive, provides an overview of current practices regarding screening and diagnosis of HF in CKM, in relation to its most frequent causes and challenges posed by the CKM continuum. Starting from current guideline recommendations, we highlighted strengths and pitfalls of “gold standard” biomarkers and briefly explored emerging biomarkers. Future research with well-designed prospective studies, exploring diverse populations and with less heterogeneity when reporting outcomes, will help us achieve the goal of reducing global CVD burden.

## Figures and Tables

**Table 1:** Overview of relationship between natriuretic peptides and obesity.

Author	Study Type	Study Population	Main Findings	Reference
Cheng et al. 2011	Observational	Community-based individuals (participants from Framingham Heart Study cohort)	logNT-proBNP levels are inversely associated with visceral adipose tissue (*p* < 0.001) logNT-proBNP levels remain inversely associated with visceral adipose tissue even after adjustment for BMI and waist circumference (beta −0.119, *p* < 0.001) logBNP is inversely associated with visceral adipose tissues even in individuals without obesity (BMI < 30 kg/m^2^)	[75]
Suthahar et al. 2018	Observational	Community-based individuals (participants from Prevention of Renal and Vascular End-Stage Disease (PREVEND))	Mean NT-proBNP was significantly higher in females (median, IQR: 50.5, 28.2–87.0 vs. 24.3, 10.1–54.6 ng/L, *p* < 0.001) NT-proBNP levels were lower with increase in weight but not associated with BMI Increase in BMI was associated with higher NT-proBNP levels in males (*p* < 0.001)	[80]
Ndumele et al. 2016	Observational	Community-based individuals (participants from ARIC study cohort)	Overall NT-proBNP levels were inversely associated with BMI (Pearson correlation coefficient −0.10, *p* < 0.001) Risk of developing incident HF was higher with increasing BMI at the same NT-proBNP range Lower NT-proBNP levels were associated with lower risk of incident HF across the BMI spectrum	[81]
Krauser et al. 2005	Observational	Patients presenting with acute dyspnea	Patients with overweight and obesity were more likely to have lower circulating NPs levels compared to patients with normal BMI (*p* < 0.001) Patients with overweight and obesity had lower odds of having NT-proBNP levels below cut-off compared to BNP	[82]
Vaishnav et al. 2020	Observational	Patients with HFpEF hospitalized for acute HF	Patients in the highest BMI category (≥40.0 kg/m^2^) represented 52% of the study group and displayed lower admission NT-proBNP compared to patients in lower BMI categories 13% of patients admitted for acute HF had “normal” NT-proBNP at admission (<125 pg/mL)	[83]
Verbrugge et al. 2022	Observational	Patients undergoing diagnostic work-up for dyspnea	157 of 420 patients diagnosed with HFpEF by invasive testing displayed NT-proBNP below guideline-recommended cut-off (<125 pg/mL) Normal NT-proBNP HFpEF was associated with less severe structural and functional abnormalities Lower NT-proBNP predicted fewer deaths and hospitalizations compared to higher NT-proBNP (HR: 0.38, 95% CI: 0.22–0.65)	[86]

Summary of findings from studies exploring the relationship between natriuretic peptides and obesity. Abbreviations: BMI = body mass index; CI = confidence interval; HFpEF = heart failure with preserved ejection fraction; IQR = interquartile range; log = logarithm; NT-proBNP = N-terminal pro-B-type natriuretic peptide.

**Table 2:** Emerging biomarkers in CKM syndrome.

Biomarker	Mechanism	Clinical Implications	References
hs-CRP/IL-6	IL-6 inflammation pathway	Independent predictors of MACE and mortality hs-CRP: predictor of incident HF IL-6: predictor of incident HFpEF	[98,99,100,102]
sST2	Profibrotic effects due to IL-33 blockade	Predictor of mortality in acute and chronic HF Predictor of incident HF Potentially influenced to lesser extent by eGFR	[93,105,106,107]
VCAM-1/ICAM-1	Endothelial dysfunction	Predictors of incident ASCVD VCAM-1: predictor of incident HF and HF phenotypes (HFpEF) Predictors of atrial myopathy and incident AF Predictors of worse outcome in HF	[113,114,115,116,117,118]
GDF-15	Profibrotic, proinflammatory	Predictor of incident ASCVD Predictor of incident HF Predictor of mortality in ASCVD and HF	[121,122,123,125,126]

Overview of emerging biomarkers and their clinical utility across the CKM spectrum. Abbreviations: AF = Atrial fibrillation; ASCVD = Atherosclerotic cardiovascular disease; eGFR = estimated Glomerular Filtration Rate; HF = Heart failure; HFpEF = Heart Failure with Preserved Ejection Fraction; hs-CRP = High-sensitivity C-reactive protein; ICAM-1 = Intercellular Adhesion Molecule-1; IL-6 = Interleukin-6; IL-33 = Interleukin-33; MACE = Major adverse cardiovascular events; sST2 = Soluble suppression of tumorigenicity-2; VCAM-1 = Vascular Cell Adhesion Molecule-1.

## Data Availability

No new data were created or analyzed in this study.

## Abbreviations

The following abbreviations are used in this manuscript:

CKM	Cardiovascular–kidney–metabolic	
AHA	American Heart Association	
CVD	Cardiovascular disease	
BMI	body mass index	
MetS	metabolic syndrome	
CKD	chronic kidney disease	
HF	heart failure	
ASCVD	atherosclerotic cardiovascular disease	
AF	atrial fibrillation	
NHANES	National Health and Nutrition Examination Survey	
CAD	coronary artery disease	
T2DM	type 2 diabetes mellitus	
LVEF	left ventricular ejection fraction	
HFrEF	heart failure with reduced ejection fraction	
HFmrEF	heart failure with mildly reduced ejection fraction	
HFpEF	heart failure with preserved ejection fraction	
NPs	natriuretic peptides	
ESC	European Society of Cardiology	
NT-proBNP	N-terminal pro-B-type natriuretic peptide	
eGFR	estimated glomerular filtration rate	
HbA1c	glycated hemoglobin	
Lp(a)	lipoprotein(a)	
apoB	apolipoprotein B	
hs-CRP	high sensitivity C-reactive protein	
LDL	low-density lipoprotein	
VLDL-C	very-low-density cholesterol	
HDL	high-density lipoprotein	
MACE	major adverse cardiovascular events	
CT	computed tomography	
CAC	coronary artery calcium	
CTA	computed tomography angiography	
PCP-HF	Pooled Cohort Equations to Prevent HF	
uACR	urine albumin/creatinine ratio	
MESA	Multi-Ethnic Study of Atherosclerosis	
BNP	B-type natriuretic peptide	
NPR-A	natriuretic peptide receptor-A	
NPR-B	natriuretic peptide receptor-B	
NPR-C	natriuretic peptide receptor-C	
cGMP	cyclic guanosine monophospate	
PRIDE	Pro-BNP investigation of dyspnea in the emergency department	
NT-ANP	N-terminal atrial natriuretic peptide	
AUC	Area under the curve	
ROC	Receiver Operating Characteristics	
MACE	major adverse cardiac events	
ADA	American Diabetes Association	
ARIC	Atherosclerosis Risk in Communities	
NRI	net reclassification improvement	
CRIC	Chronic Renal Insufficiency Cohort	
CRP	C-reactive protein	
IL-6	interleukin-6	
TNF-α	tumor necrosis factor α	
PREVEND	Prevention of Renal and Vascular End-Stage Disease	
sST2	Soluble suppression of tumorigenicity 2	
IL-33	interleukin-33	
MR-proADM	mid-regional pro-adrenomedullin	
NYHA	New York Heart Association	
VCAM-1	vascular cellular adhesion molecule-1	
ICAM-1	intracellular adhesion molecule-1	
GLS	global longitudinal strain levels	
CMR	cardiac magnetic resonance	
GDF-15	growth-differentiation factor-15	
LVH	left ventricular hypertrophy	
LVSD	left ventricular systolic dysfunction	
HOMA-IR	homeostatic model assessment of insulin resistance	
TyG	Triglyceride-Glucose Index	
TyG-BMI	Triglyceride-Glucose Index and Body Mass Index Product	
TyG-WC	TyG-waist circumference Index	
SCFAs	short-chain fatty acids	
TMAO	trimethylamine N-oxide	
DI-GM	dietary index for gut microbiota	
